# Associations of Serum Urea With Neutrophil-to-Lymphocyte Ratio in Lung Cancer Patients

**DOI:** 10.7759/cureus.106252

**Published:** 2026-04-01

**Authors:** Olivian Savencu, Carla Bianca Vulturar, Horia Dan Liscu, Nicolae I Verga

**Affiliations:** 1 Radiation Oncology, Carol Davila University of Medicine and Pharmacy, Bucharest, ROU; 2 Radiotherapy, Carol Davila University of Medicine and Pharmacy, Bucharest, ROU

**Keywords:** immune-metabolic interaction, lung cancer, neutrophil-to-lymphocyte ratio (nlr), serum urea, systemic inflammation

## Abstract

Systemic inflammation and metabolic alterations frequently coexist in lung cancer, yet their interrelationship remains incompletely understood. Serum urea is routinely measured in clinical practice but is rarely examined in relation to immune-inflammatory indices. We conducted a retrospective cross-sectional analysis of lung cancer patients with available serum urea and complete blood count data. Associations between serum urea and inflammatory immune indices, including the neutrophil-to-lymphocyte ratio (NLR), were evaluated using non-parametric correlation analyses. Multivariable regression and exploratory mediation analyses were performed to assess the contribution of lymphocyte count to observed associations. Eighty-three patients were included in the final analysis. Serum urea was positively associated with NLR and inversely associated with absolute lymphocyte count. Serum creatinine did not demonstrate a comparable association with NLR. Exploratory mediation analysis suggested that lymphocyte count partially mediated the association between serum urea and NLR. Sensitivity analyses excluding explicitly treated patients and additional adjustment for hepatic biochemical markers did not materially alter the findings. In patients with lung cancer, higher serum urea levels are associated with systemic immune dysregulation, reflected by elevated NLR and lower lymphocyte counts. These findings suggest that serum urea may serve as an integrative marker of metabolic-immune coupling beyond its conventional interpretation as a marker of renal function. Further longitudinal studies are warranted to clarify the clinical implications of this association.

## Introduction

Lung cancer is frequently accompanied by systemic inflammation, which reflects complex interactions between tumor biology and host responses [1]. This inflammatory state has been associated with disease burden, functional decline, and adverse outcomes, and has therefore attracted interest as a source of accessible clinical biomarkers [2]. Among these, blood-based inflammatory indices derived from routine laboratory testing are increasingly used due to their low cost, availability, and reproducibility [3].

The neutrophil-to-lymphocyte ratio (NLR) is one of the most widely studied inflammatory markers in oncology [4]. Elevated NLR has been associated with systemic inflammatory activation and relative suppression of adaptive immunity, and has been linked to unfavorable clinical outcomes across multiple cancer types, including lung cancer [5]. Despite its broad use, NLR remains a composite index, and the upstream metabolic and physiological factors that influence its variability in lung cancer patients are not fully understood.

Serum urea is routinely measured in clinical practice and is commonly interpreted as a marker of renal function. However, urea also reflects whole-body nitrogen metabolism and protein catabolism, processes that may be altered in chronic inflammatory states [6]. In cancer, systemic inflammation and metabolic stress can promote muscle proteolysis and increased hepatic nitrogen turnover, potentially influencing circulating urea levels independently of glomerular filtration [7,8]. Despite this biological plausibility, the relationship between serum urea and inflammatory immune indices such as NLR has received limited attention in lung cancer populations.

Understanding whether serum urea is associated with inflammatory immune markers may help clarify links between metabolic stress and immune imbalance in lung cancer. Moreover, examining the role of lymphocyte count in this association may provide insight into how metabolic and immune processes converge within the circulating blood compartment [9]. Therefore, the primary objective of this study was to evaluate the association between serum urea and inflammatory immune indices, particularly the ratio NLR, in a cohort of lung cancer patients predominantly assessed prior to oncologic treatment. Secondary objectives included assessing the relationship between serum urea and individual leukocyte components, particularly absolute lymphocyte count, and exploring whether lymphocyte count mediates the association between serum urea and NLR through multivariable and exploratory mediation analyses.

## Materials and methods

Study design

This was a retrospective cross-sectional observational study that included adult patients with histologically confirmed lung cancer identified from the institutional medical records of MedEuropa Oradea, a tertiary oncology center in Oradea, Romania. Data were collected retrospectively from January 2016 to January 2026. The study was approved by MedEuropa Oradea (approval number: Med039/22.01.2026) and was conducted in accordance with the Declaration of Helsinki and applicable national regulations governing retrospective observational research. 

Study population

Patients were included if serum urea and complete blood count parameters required for NLR calculation were available from the same laboratory assessment. Patients without serum urea measurements or with missing neutrophil or lymphocyte counts were excluded due to the inability to compute inflammatory immune indices. No exclusions were made based on lung cancer histological subtype, disease stage, or treatment status. Treatment timing at the time of blood sampling was explicitly documented as pre-treatment in a subset of patients and as during or after oncologic treatment in a small number of cases; treatment timing was not documented for all patients.

A total of 113 patients were screened. Serum urea measurements were available for 86 patients, of whom 83 also had complete blood count data obtained at the same time point, including absolute neutrophil and lymphocyte counts, allowing calculation of the NLR. These 83 patients constituted the final analytic cohort. 

Data collection

Laboratory data were obtained from routine blood tests performed as part of standard clinical care. All laboratory analyses were performed in a certified clinical laboratory using standardized automated analyzers according to the manufacturer’s protocols. Variables analyzed included serum urea, serum creatinine, hemoglobin, absolute neutrophil count, and absolute lymphocyte count. All laboratory values were derived from a single blood sample per patient. The NLR was calculated as the ratio of absolute neutrophil count to absolute lymphocyte count. Absolute lymphocyte count was also analyzed independently due to its relevance to adaptive immune function. Hepatic biochemical markers, including aspartate aminotransferase, alanine aminotransferase, gamma-glutamyl transferase, alkaline phosphatase, and bilirubin, were used as secondary control variables in sensitivity analyses where available.

Statistical analysis

Continuous variables were summarized using distribution-appropriate descriptive statistics, and associations between continuous variables were evaluated using Spearman's rank correlation coefficient. Multivariable linear regression analyses were conducted with NLR as the dependent variable and serum urea, age, sex, and serum creatinine as independent variables, using complete-case data.

Exploratory mediation analyses were performed using standard linear regression-based approaches implemented in Python (e.g., statsmodels), with serum urea as the independent variable, NLR as the dependent variable, and absolute lymphocyte count as a potential mediator. Sensitivity analyses included exclusion of laboratory assessments obtained during or after oncologic treatment, as well as additional adjustment for hepatic biochemical markers. All statistical analyses were performed in Python using standard scientific libraries. A two-sided p-value < 0.05 was considered statistically significant.

## Results

Patient characteristics

A total of 83 lung cancer patients were included in the final analysis. Baseline demographic and laboratory characteristics of the study population are summarized in Table 1. The cohort had a mean age of 64.4 years and was predominantly male. Serum urea, serum creatinine, hemoglobin, and complete blood count parameters required for the calculation of inflammatory immune indices were available for all included patients.

**Table 1:** Baseline demographic and laboratory characteristics of the study population (N = 83) Statistical significance was defined as p < 0.05 IQR: interquartile range

Variable	Value
Demographic characteristics	
Age (years), mean ± SD	64.41 ± 7.11
Sex (male), n (%)	63 (75.9%)
Renal and metabolic parameters, median (IQR)	
Serum urea (mg/dL)	30.00 (23.27–40.68)
Serum creatinine (mg/dL)	0.76 (0.64–0.90)
Hematologic parameters	
Hemoglobin (g/dL), mean ± SD	12.34 ± 1.71
Neutrophil count (cells/µL), median (IQR)	5100 (3465–6765)
Lymphocyte count (cells/µL), median (IQR)	1340 (990–1775)
Neutrophil-to-lymphocyte ratio, median (IQR)	3.69 (2.63–5.61)
Clinical characteristics, n (%)	
Explicitly documented pre-treatment at blood sampling	63 (75.9%)

Treatment timing at the time of blood sampling was explicitly documented as pre-treatment in 63 patients (75.9%) and as during or after oncologic treatment in seven patients (8.4%). For the remaining patients, treatment timing was not documented.

Associations between serum urea and inflammatory immune indices

**Figure 1:**
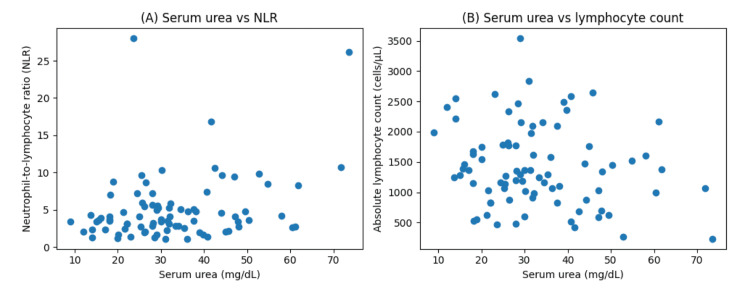
Association of serum urea with inflammatory immune indices in lung cancer patients (N = 83) (A) Scatter plot showing the association between serum urea levels and NLR. (B) Scatter plot showing the association between serum urea levels and absolute lymphocyte count. Each point represents one patient. Associations were evaluated using Spearman's rank correlation coefficient (ρ). NLR: neutrophil-to-lymphocyte ratio

Serum urea demonstrated a positive association with the NLR (Spearman correlation: ρ = 0.34, p = 0.0017; Figure 1A). In contrast, serum creatinine did not show a significant association with NLR, suggesting that the observed relationship was not driven by renal dysfunction alone.

When examining individual leukocyte components, serum urea was inversely associated with absolute lymphocyte count (Spearman correlation: ρ = −0.39, p = 0.0003; Figure 1B). The association between serum urea and absolute neutrophil count was weaker and not consistently observed. These findings suggest that the observed urea-NLR relationship may be primarily influenced by variation in lymphocyte counts rather than neutrophil expansion.

Multivariable linear regression analyses adjusting for age, sex, and serum creatinine were performed on 78 patients with complete data for all variables included in the model. Serum urea remained independently associated with NLR (B = 0.086, SE = 0.042, t = 2.08, p = 0.041; 95% CI 0.003-0.169). Age, sex, and serum creatinine were not significantly associated with NLR in the adjusted model. The overall regression model demonstrated borderline statistical significance (F(4,73) = 2.47, R² = 0.119, adjusted R² = 0.071, p = 0.052). These findings suggest that the association between serum urea and NLR persists after adjustment for demographic variables and renal function markers, although the strength of the model was modest (Table 2).

**Table 2:** Evaluation of the association between serum urea and NLR, adjusted for age, sex, and serum creatinine. Data are presented as unstandardized regression coefficients (B), standard errors (SE), t values, p values, and 95% confidence intervals. The model was adjusted for age, sex, and serum creatinine. Statistical significance was defined as p < 0.05. Regression analyses included 78 patients with complete data for all model variables. NLR: neutrophil-to-lymphocyte ratio

Variable	B	SE	t	P	95% CI
Serum urea	0.086	0.042	2.08	0.041	0.003–0.169
Age	-0.069	0.076	-0.91	0.365	-0.220–0.082
Sex (male)	-0.410	1.235	-0.33	0.741	-2.872–2.052
Serum creatinine	2.808	2.394	1.17	0.245	-1.964–7.579

In multivariable linear regression analyses adjusting for age, sex, and serum creatinine, serum urea remained independently associated with NLR. Exploratory mediation analysis further suggested that absolute lymphocyte count partially mediated the association between serum urea and NLR, as inclusion of lymphocyte count in regression models attenuated the strength of the urea-NLR association.

Sensitivity analyses excluding laboratory assessments obtained during or after oncologic treatment yielded associations consistent in direction with the primary analyses. Additional adjustment for hepatic biochemical markers did not materially alter the observed relationships between serum urea and inflammatory immune indices.

## Discussion

In this retrospective cross-sectional study of lung cancer patients, we identified a consistent association between serum urea and systemic inflammatory immune indices, particularly the NLR. This association was driven primarily by lower absolute lymphocyte counts rather than by neutrophil expansion, suggesting that serum urea reflects a broader metabolic-immune coupling state rather than renal dysfunction alone.

Although serum urea is conventionally interpreted as a marker of renal function, it is also influenced by protein catabolism and systemic inflammation, both common features in malignancy [10]. The absence of a comparable association between serum creatinine and NLR in this cohort argues against a purely renal explanation. Instead, elevated urea may represent a downstream metabolic signature of sustained inflammatory and catabolic processes associated with cancer [11].

The inverse relationship between serum urea and lymphocyte count further supports this interpretation. Lymphopenia is a well-recognized feature of cancer-related immune dysregulation, and exploratory mediation analysis suggested that lymphocyte count partially mediated the urea-NLR association [12]. While causal inference is not possible in this cross-sectional design, these findings are consistent with known immune-metabolic interactions and highlight lymphocyte dynamics as a key contributor to composite inflammatory indices.

From a clinical standpoint, serum urea is a routinely available and inexpensive laboratory parameter. Its association with immune dysregulation may therefore have practical relevance for the integrative assessment of lung cancer patients, particularly in settings where advanced immune profiling is unavailable. The observed associations were robust in sensitivity analyses excluding explicitly treated patients and after adjustment for hepatic biochemical markers, suggesting that treatment effects and hepatic dysfunction alone do not account for the findings.

Limitations

Several limitations should be acknowledged. The retrospective and cross-sectional design precludes assessment of temporal relationships or causality, and laboratory parameters were evaluated at a single time point. Treatment timing and disease stage were not available for all patients, limiting adjustment for clinical heterogeneity. Additionally, detailed data on comorbidities, nutritional status, inflammatory markers such as C-reactive protein, and body composition were not systematically recorded, and residual confounding cannot be excluded. Finally, the study was conducted in a single-center cohort, which may limit generalizability. Prospective longitudinal studies are needed to evaluate dynamic changes in serum urea and immune indices in relation to treatment response and clinical outcomes. Although serum urea remained independently associated with NLR in multivariable analysis, the overall model demonstrated borderline statistical significance and explained a modest proportion of variance. These findings should therefore be interpreted as exploratory and hypothesis-generating. Validation in larger, well-characterized cohorts is necessary to confirm the robustness and clinical relevance of the observed associations.

## Conclusions

In lung cancer patients, serum urea is associated with systemic inflammatory immune indices, particularly the NLR, with this relationship driven primarily by lower absolute lymphocyte counts. These findings suggest that serum urea may reflect an underlying metabolic-immune coupling state beyond its conventional interpretation as a marker of renal function. Given its routine availability, serum urea may have value as an integrative biomarker in the assessment of cancer-related immune dysregulation. Further longitudinal studies are warranted to clarify the clinical implications of this association.
